# The prognostic value of the lymph node ratio for local advanced gastric cancer patients with intensity-modulated radiation therapy and concurrent chemotherapy after radical gastrectomy in China

**DOI:** 10.1186/s13014-020-01687-0

**Published:** 2020-10-14

**Authors:** Yongqiang Yang, Yifu Ma, Xiaoyong Xiang, Pengfei Xing, Yongyou Wu, Liyuan Zhang, Ye Tian

**Affiliations:** 1 Department of Radiotherapy and Oncology, The Second Affiliated Hospital of Soochow University, San Xiang Road No. 1055, Suzhou, 215004 China; 2 Institute of Radiotherapy and Oncology, Soochow University, Suzhou, 215004 China; 3 Suzhou Key Laboratory for Radiation Oncology, Suzhou, 215004 China; 4 Suzhou Key Laboratory for Combined Radiotherapy and Immunotherapy of Cancer, Suzhou, 215004 China; 5 Department of Radiation Oncology, National Cancer Center/National Clinical Research Center for Cancer/Cancer Hospital and Shenzhen Hospital, Chinese Academy of Medical Sciences and Peking Union Medical College, Shenzhen, 518116 China; 6 Department of Gastrointestinal Surgery, The Second Affiliated Hospital of Soochow University, Suzhou, 215004 China

**Keywords:** Gastric cancer, Radiotherapy, Lymph node ratio, Chemoradiation, China

## Abstract

**Background:**

Nearly 50% of new gastric cancer cases and gastric cancer-related deaths worldwide occur in China. No global consensus has been reached about the optimal management of locally advanced gastric cancer. Although the Guidelines for the Diagnosis and Treatment of Gastric Cancer from the National Health Commission of China, which has been updated three times since 2010, explicitly emphasize the necessity of adjuvant chemoradiation, few clinical institutions in China routinely adhere to the recommended radiotherapy guidelines. This study aimed to examine the efficacy, in terms of locoregional control and long-term survival, and the safety of adjuvant radiotherapy using intensity-modulated radiation therapy (IMRT) with concurrent and adjuvant fluoropyrimidine-based chemotherapy for gastric cancer.

**Methods:**

This was a retrospective evaluation of 156 patients with high-risk gastric cancer who underwent adjuvant chemoradiotherapy between September 2008 and May 2019. The prescribed planning target volume median dose was 45 Gy in 1.8 Gy daily fractions, and all patients received concurrent and adjuvant fluoropyrimidine-based chemotherapy. Locoregional control, distant metastasis, and overall survival rates were estimated. Clinicopathological characteristics and patterns of failure were retrospectively reviewed to identify factors associated with survival and recurrence.

**Results:**

The median follow-up duration was 56 months (range 3–130 months) for all patients. Of the patients, 11 (7.1%) were lost to follow-up, and 49 (31.4%) and 104 (66.7%) had stage II or III disease according to the eighth edition of the American Joint Committee on Cancer tumor-node-metastasis staging criteria. The frequencies of acute grade 3 or 4 gastrointestinal and hematological toxicity were 9.6% and 10.9%, respectively. In total, 152 patients (97.4%) completed the entire chemoradiation regimen. No toxicity-related deaths occurred. Nineteen patients (12.2%) had locoregional recurrence, 26 (16.7%) had distant metastases, and 12 (7.7%) had peritoneal metastasis. The overall survival (OS) rates were 83.5%, 65.0%, and 59.5%, while the disease-free survival rates were 75.1%, 61.0%, and 55.6% at 1, 3, and 5 years, respectively. In the multivariate analysis, age, pathological T stage and lymph node ratio (LNR) were found to be independent predictors of OS.

**Conclusion:**

Postoperative concomitant IMRT and chemotherapy were well tolerated, with acceptable toxicities and encouraging locoregional tumor control and long-term survival. The LNR can be used as an important prognostic indicator for OS. Adjuvant chemoradiotherapy should be considered for all patients with a high risk of locoregional recurrence, especially in China.

## Background

Gastric cancer is the fifth most common malignant tumor and third most common in terms of mortality worldwide [1]. Nearly half of the global cases occur in China [2]. The morbidity and mortality rates of gastric cancer rank second among all types of cancer in China [3]. Surgical resection is the primary treatment for nonmetastatic gastric cancer. Notably, there was a marked overall increase in gastric cancer survival from 2003 to 2015 in China [4]. However, in patients with localized or locally advanced disease, the prognosis remains dismal after surgery and adjuvant chemotherapy, with more than 60% of patients relapsing, especially within 2 years after surgery [5]. Although the benefit of this approach following D0 or D1 lymph node dissection has not been investigated in randomized clinical trials, adjuvant chemoradiotherapy is considered the standard of treatment for this subpopulation of gastric cancer patients, as demonstrated by the results of INT-0116 [6]. No global consensus has been reached about the optimal management of locally advanced gastric cancer [7]. The benefit of adjuvant chemoradiotherapy for patients with D2 lymph node dissection in locally advanced gastric cancer remains controversial [8]. The results of the ARTIST trial for a subgroup of patients with node-positive gastric cancer suggested that adjuvant chemoradiotherapy had a significant effect on both disease-free survival (DFS) and locoregional recurrence-free survival (LRRFS) [9, 10]. In China, the treatment and surgical outcomes of gastric cancer vary greatly across different regions. Current guidelines in China recommend D2 radical resection as the preferred approach for improving the long-term survival of patients with gastric cancer. However, not all medical institutions have the capacity to perform standard D2 lymphadenectomy. Studies have shown that even at leading treatment centers, nearly 50% of advanced gastric cancer patients do not undergo standard D2 resection in China [11]. The percentage could be even lower at other centers in China. As radiotherapy is a local therapy that can complement surgery, it is important to adopt adjuvant chemoradiation to treat gastric cancer in China [12]. Evidence from patients after D2 lymphadenectomy and adjuvant chemoradiotherapy (CRT) is still insufficient, especially with respect to risk factors for different types of failure. Therefore, the purpose of this study was to characterize the patterns of failure in patients after radical surgery and adjuvant CRT. By investigating the relationship between clinicopathologic factors and recurrence, additional evidence may be provided for the selection of patients based on the predicted risk of each recurrence pattern.

## Methods

### Patient identification

Patients with curatively resected gastric carcinoma who received postoperative CRT between September 2008 and May 2019 were retrospectively identified. Patients who met the following eligibility criteria were included: underwent R0 gastrectomy and ≥ D1+ lymphadenectomy; had no clinical evidence of distant metastasis (M0) or peritoneal metastasis; received postoperative CRT; followed up regularly after treatment; and had complete medical record data available. Patients who met the following exclusion criteria were not included: received preoperative chemotherapy or radiotherapy or had inadequate function of the liver, kidneys, or any other major organs. All patients in the study signed informed consent forms.

### Treatment

The surgical requirement for eligibility was radical resection. Radiotherapy was delivered using intensity-modulated radiation therapy (IMRT). Patients were treated with a median dose of 45 Gy (range 41.4–50.4 Gy) delivered at 1.8 Gy/fraction. The radiation target volumes encompassed the tumor bed, anastomosis site, duodenal stump, and selected regional lymph nodes (LNs). The tumor bed of patients with pT1 and pT2 M0 gastric cancer is not considered to be irradiated. The selection of regional LNs, including perigastric, celiac, splenic, hepatoduodenal or hepatic-portal, pancreaticoduodenal and paraaortic LNs, depended on the location of the tumor. Chemotherapy was administered at 3–8 weeks after surgery, followed by chemoradiation beginning at 8–18 weeks after surgery.

### Follow-up

After the completion of adjuvant CRT, regular follow-up was conducted in accordance with the institutional surveillance strategy, including medical history, physical examination, serum biochemical, tumor biomarkers, CT scans of the chest, abdomen and pelvis (or positron-emission tomographic scans if necessary) and endoscopy at each visit. Patients were followed up every 3 months for the first 2 years, every 6 months until 5 years and yearly thereafter.

### Recurrence analyses

Local recurrence was defined as recurrence at the anastomosis site, duodenal stump, tumor bed, or remnant stomach. Regional recurrence was defined as recurrence at regional LNs such as the perigastric, porta hepatis, peripancreatic and paraaortic LNs. Peritoneal dissemination was considered to include metastasis of the peritoneum, colorectum, ovary, and ureter. Distant metastasis was defined as metastasis to a distant organ such as liver, bone, or lung or lymph node recurrence, except for regional LNs. All of the patients’ medical records were reviewed, and any relapse or metastasis was documented. If two or more failure sites developed at the same time, they were counted separately. Overall survival (OS) was defined as the time from surgery to death, including tumor-specific death or death from any other cause. Disease-free survival (DFS) was considered as the time from surgery to initial recurrence or death, and local failure-free survival (LFFS)/regional failure-free survival (RFFS)/peritoneal failure-free survival (PFFS)/distant failure-free survival (DFFS) were defined as the time from surgery to local/regional/peritoneal/distant failure.

### Statistical analysis

Data were recorded as categorical and continuous variables. Actuarial curves of LFFS, RFFS, locoregional failure-free survival (LRFFS), PFFS, DFFS, DFS, and OS were plotted using Kaplan–Meier estimates. Univariate and multivariate Cox regression analyses were used to identify prognostic factors. The variables included age, sex, operation hospital, operative approach, location of primary tumor, pathologic types, Lauren's classification, number of dissected LNs, number of positive LNs, pathologic T stage, pathologic N stage, pathologic tumor-node-metastasis (TNM) stage, lymphovascular invasion (LVI), perineural invasion (PNI), concurrent radiochemotherapy regimen, and adjuvant chemotherapy regimen. All p-values were two-sided, and a *p* value < 0.05 was considered statistically significant. All statistical analyses were performed using IBM SPSS v. 25.0 (SPSS Inc., Chicago, IL).

## Results

### Study population and clinicopathological characteristics

**Fig. 1:**
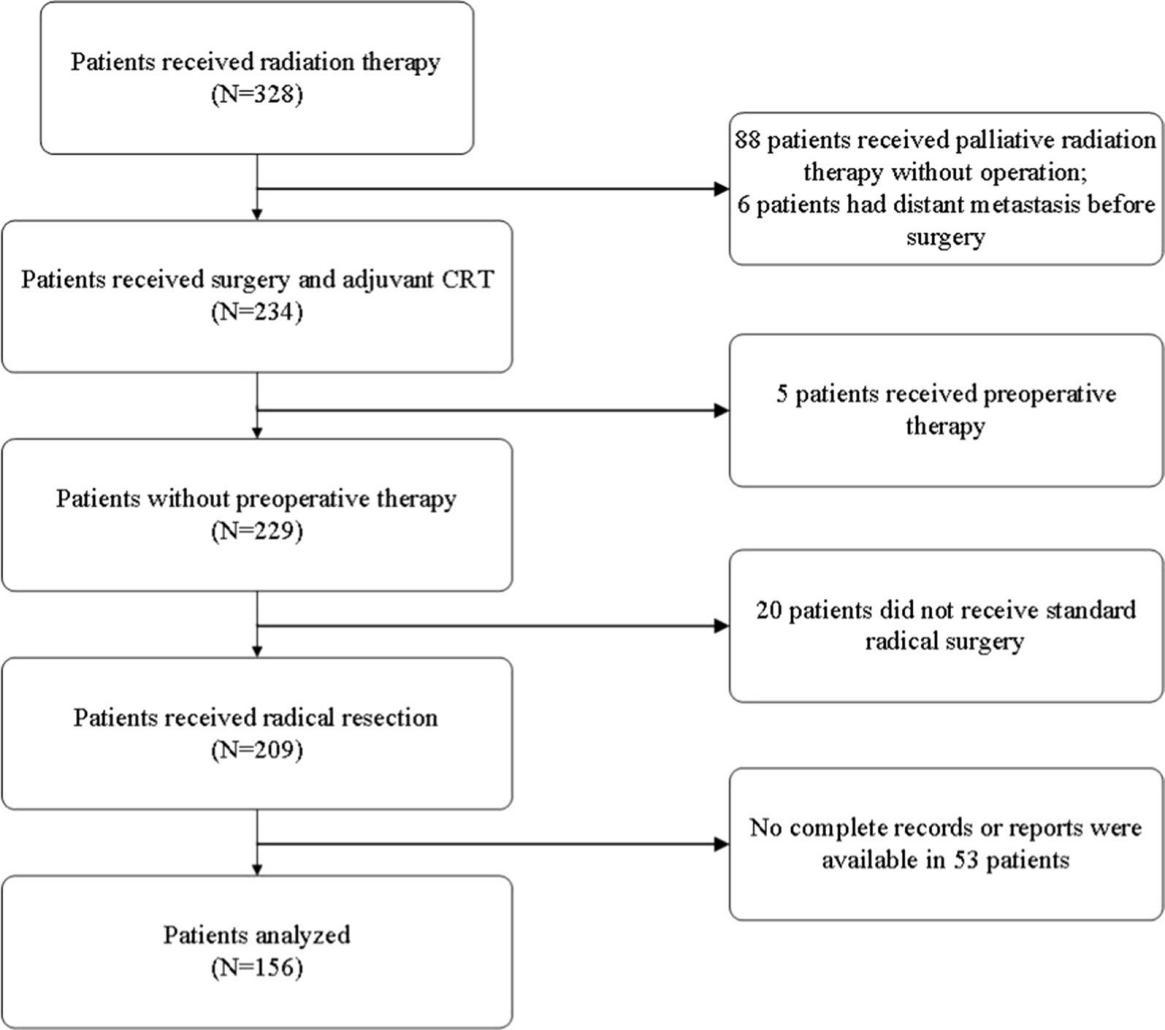
Flow diagram of patient selection according to the eligibility criteria and exclusion criteria

A total of 328 patients with gastric cancer received radiation therapy. Of these, 172 patients were excluded due to the following reasons: 88 received palliative radiation therapy without surgery, 6 had distant metastasis before gastrectomy, 5 received preoperative therapy, 20 did not receive standard radical surgery, and 53 did not have complete pathologic reports or medical record data. Ultimately, 156 patients met the criteria and were included in the analysis (Fig. 1). The first patient underwent radical surgery in July 2008, and the last patient was treated in April 2019. The loss to follow-up rate was 7.1% (11 of 156 patients).

The patient characteristics and surgical results are listed in Table 1. Nearly three-quarters (73.1%) of the 156 patients were male. The median age was 60 years (range 27–76 years). Most of the enrolled patients were in pathological stage III (66.0%), and nearly half (41.7%) of the enrolled patients had N3 disease. The median number of dissected and positive LNs was 19 and 5, respectively (range 5–56 and 0–47).

**Table 1:** Characteristics of the patients (N = 156)

Characteristics	No. of patients (%)
Age (years)	
Median	60 (27–76)
≤ 40	6 (3.8%)
41–65	114 (73.1%)
≥ 66	36 (23.1%)
Sex	
Male	114 (73.1%)
Female	42 (26.9%)
Operative hospital	
Our hospital	93 (59.6%)
Another hospital	63 (40.4%)
Operative approach	
Proximal subtotal gastrectomy	24 (15.4%)
Distal subtotal gastrectomy	69 (44.2%)
Total gastrectomy	53 (34.0%)
Gastrectomy combined with resection of other organs	10 (6.4%)
Location of primary tumor	
Upper 1/3	39 (25%)
Middle 1/3	33 (21.2%)
Lower 1/3	69 (44.2)
Total stomach	15 (9.6%)
Pathologic types	
Well to moderately differentiated adenocarcinoma	52 (33.3%)
Poorly differentiated adenocarcinoma	82 (52.6%)
Mucinous adenocarcinoma	9 (5.8%)
Signet ring cell adenocarcinoma	12 (7.7%)
Neuroendocrine degeneration	1 (0.6%)
Lauren's classification	
Intestinal type	63 (40.4%)
Diffuse type	86 (55.1%)
Mixed unclassified	7 (4.5%)
No. of dissected LNs	
Median	19 (5–56)
< 15	52 (33.3%)
≥ 15	104 (66.7%)
No. of positive LNs	
Median	5 (0–47)
LNR = 0	24 (15.3%)
0 < LNR < 0.3	58 (37.2%)
0.3 ≤ LNR < 0.7	53 (34.0%)
0.7 ≤ LNR	21 (13.5%)
Pathologic T stage	
T1	8 (5.1%)
T2	19 (12.2%)
T3	73 (46.8%)
T4a	31 (19.9%)
T4b	25 (16.0%)
Pathologic N stage	
N0	23 (14.7%)
N1	30 (19.2%)
N2	37 (23.7%)
N3a	53 (34.0%)
N3b	13 (8.3%)
Stage	
IB	3 (1.9%)
IIA	26 (16.7%)
IIB	23 (14.7%)
IIIA	37 (23.7%)
IIIB	40 (25.6%)
IIIC	27 (17.3%)
Lymphovascular invasion (LVI)	
Negative	74 (47.4%)
Positive	82 (52.6%)
Perineural invasion (PNI)	
Negative	83 (53.2%)
Positive	73 (46.8%)
Concurrent chemotherapy regimen	
Capecitabine	88 (56.4%)
S-1	22 (14.1%)
Others	46 (29.5%)
Adjuvant chemotherapy regimen	
XELOX	76 (48.7%)
SOX	27 (17.3%)
FLOFOX	13 (8.3%)
EOF	14 (9.0%)
FLOT	4 (2.6%)
Others	22 (14.1%)

### Treatment delivery

Patients were treated with a median dose of 45 Gy (range 41.4–50.4 Gy), with 1.8 Gy daily fractions. The median duration of radiation was 35 days (range 30–45 days). All enrolled patients received adjuvant chemotherapy before or after radiotherapy. The chemotherapy regimens included the following: CAPOX (capecitabine and oxaliplatin) (76, 48.7%); SOX (S-1 and oxaliplatin) (21, 13.5%); EOF (epirubicin, oxaliplatin and 5-FU) (14, 9.0%); FOLFOX (oxaliplatin and 5-FU) (13, 8.3%) and other agents.

### Survival and prognostic factors

**Fig. 2:**
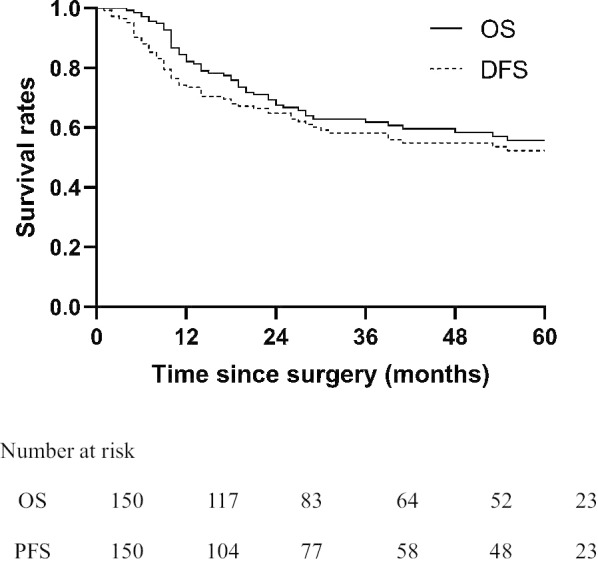
Kaplan–Meier estimate of OS and DFS. *DFS* disease-free survival, *OS* overall survival

The patients were followed up until May 2019, and the median follow-up period was 56.0 months (range 1–130 months). During the follow-up period, a total of 57 (36.5%) deaths occurred, and 44 patients (28.2%) experienced relapse. The 1-, 3-, and 5-year OS and 1-, 3-, and 5-year DFS were 83.5%, 65.0%, and 59.5% and 75.1%, 61.0%, and 55.6%, respectively (Fig. 2). Univariate analysis identified age, pathologic types, Lauren's classification, lymph node ratio (LNR), perineural invasion, pathological T stage, N stage and pTNM stage as related to OS, whereas further multivariate analysis indicated that only age, pathological T stage and LNR were independent prognostic factors (Table 2). Lauren's classification, LNR, pathological T stage, N stage and pTNM stage were related to DFS. The results of multivariate analysis showed that pathological T stage was the only independent prognostic factor associated with DFS (Table 3).

**Table 2:** Multivariate analysis of prognosis factors associated with OS

Factor	Univariate analysis	Multivariate analysis		
		HR	95% CI	*p* value
Age	.048	2.075	1.190–3.616	.010*
Pathology	.046	1.144	0.308–4.245	.840
Lauren's classification	.045	1.751	0.509–6.022	.374
LNR	.000	2.109	1.202–3.701	.009*
Pathologic T stage	.006	1.693	1.015–2.822	.044*
Pathologic N stage	.000	0.829	0.449–1.530	.548
Stage	.021	0.886	0.286–2.748	.834
PNI	.045	1.455	0.838–2.527	.183

Bold indicates the significant values (*p < 0.05)

**Table 3:** Multivariate analysis of prognosis factors associated with DFS

Factor	Univariate analysis	Multivariate analysis		
		HR	95% CI	*p* value
Pathology	.087	0.879	0.246–3.144	.843
Lauren's classification	.042	1.699	0.509–5.671	.389
LNR	.000	1.534	0.919–2.562	.102
Pathologic T stage	.004	1.746	1.091–2.793	.020*
Pathologic N stage	.000	1.201	0.697–2.069	.510
Stage	.026	0.633	0.228–1.756	.379
PNI	.164	1.229	0.737–2.050	.428

Bold indicates the significant values (*p < 0.05)

### Adverse events

Interruption of radiation or incomplete radiation was recorded for only 4 patients. All individuals received concurrent chemotherapy, and among them, 26 (16.7%) experienced dose delay or reduction. The acute grade 3 or 4 gastrointestinal and hematological toxicity rates were 9.6% and 10.9%, respectively.

### Overall patterns of failure

**Fig. 3:**
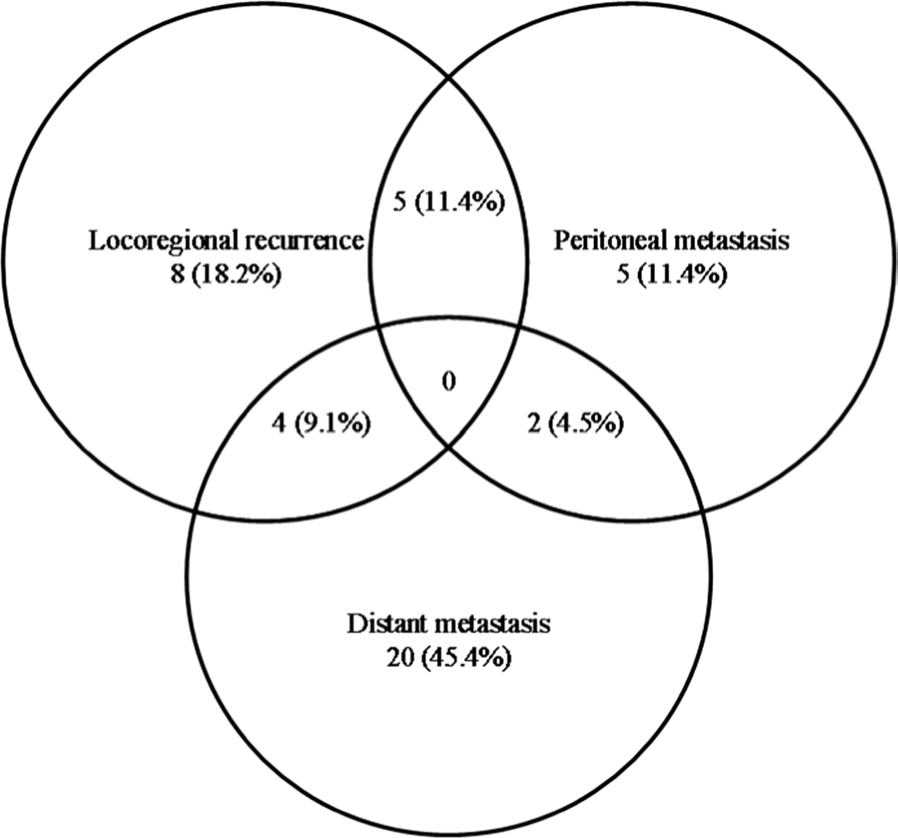
Venn diagram of the failure pattern

During the follow-up period, 44 patients (28.2%) showed relapse at 57 sites. The proportion of patients who encountered local recurrence/regional failure/peritoneal metastasis/distant metastasis in the total number of patients was 3.2/9.0/7.7/16.7%. The Venn diagram of the failure pattern is shown in Fig. 3.

Among the 44 patients who encountered relapse, single-site recurrence was noted in 31 patients (70.5%) and multisite recurrence in 13 (29.5%) patients at the time of diagnosis (Table 4). As a single pattern, distant metastasis was observed most frequently (64.5%, 20 of 31 patients). In contrast, peritoneal metastasis, regional recurrence and local failure were notably rare (16.1, 16.1 and 3.3%). As shown in Table 5, involvement of the liver, bone, lung, brain, spleen and adrenal gland as any component of metastasis occurred in 29.5% (13 of 44 patients), 20.5% (9 of 44 patients), 20.5% (9 of 44 patients), 6.8% (3 of 44 patients), 2.3% (1 of 44 patients) and 2.3% (1 of 44 patients) of cases, respectively. The most common combined pattern was regional recurrence, with distant metastasis occurring in four patients.

**Table 4:** Patterns of recurrence

Recurrence site	No. of patients	% of recurrence patients (n = 44)
Single site	31	70.5
Local recurrence	1	2.3
Regional recurrence	5	11.4
Peritoneal metastasis	5	11.4
Distant metastasis	20	45.4
Two sites	13	29.5
Local + peritoneal failure	2	4.5
Local + regional failure	2	4.5
Regional + peritoneal failure	3	6.8
Regional + distant failure	4	9.1
Peritoneal + distant failure	2	4.5
Three or more sites	0	0

**Table 5:** Recurrence sites of 44 patients

Recurrence site	No. of patients	% of recurrence patients (n = 44)	% of enrolled patients (n = 156)
Local recurrence	5	11.4	3.2
Remnant stomach	1	2.3	
Anastomosis site	4	9.1	
Regional failure	14	31.8	9.0
Peritoneal metastasis	12	27.3	7.7
Peritoneum	9	20.5	
Ovary	2	4.5	
Colorectum	1	2.3	
Distant metastasis	26	59.1	16.7
Liver	13	29.5	
Bone	9	20.5	
Lung	9	20.5	
Brain	3	6.8	
Spleen	1	2.3	
Adrenal	1	2.3	
Nonregional LNs	1	2.3	

### Survival rate for each failure pattern

**Fig. 4:**
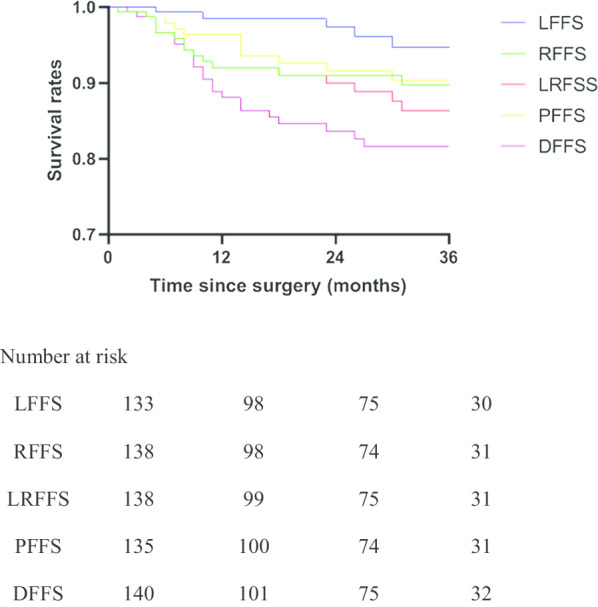
Kaplan–Meier estimate of LFFS, DFFS, RFFS, LRFFS and PFFS of patients treated with adjuvant chemoradiation. *LFFS* local failure-free survival, *DFFS* distant failure-free survival, *RFFS* regional failure-free survival, *LRFFS* locoregional failure-free survival, *PFFS* peritoneal failure-free survival

Figure 4 depicts the survival rate for each failure pattern. The 3-year survival rates are 94.8% for LFFS, 90.3% for PFFS, 89.7% for RFFS, 86.4% for LRFFS and 81.6% for DFFS. Univariate analysis revealed N stage as an influencing factor for all types of failure except peritoneal metastasis, whereas T stage affected only LRFFS. The incidence of failure increased in proportion to the N stage. In addition, LVI was associated with PFFS, and LNR was related to DFFS. Upon further multivariate Cox regression analysis, T stage was shown to be an independent risk factor for LRFFS (*p* = 0.008) and PFFS (*p* = 0.037). The independent risk factor involved in peritoneal metastasis was LVI (p = 0.031) (Tables 6, 7). We found no independent risk factor for DFFS in our study.

**Table 6:** Multivariate analysis of prognosis factors associated with locoregional recurrence

Factor	Univariate analysis	Multivariate analysis		
		HR	95% CI	*p* value
Sex	.189	0.338	0.120–0.955	.041*
Pathology	.170	6.512	1.247–34.003	.026*
LNR	.077	0.993	0.322–3.064	.990
Pathologic T stage	.037	3.096	1.341–7.149	.008*
Pathologic N stage	.022	0.800	0.328–1.950	.624
LVI	.073	2.816	0.967–8.203	.058

Bold indicates the significant values (*p < 0.05)

**Table 7:** Multivariate analysis of prognosis factors associated with peritoneal metastasis

Factor	Univariate analysis	Multivariate analysis		
		HR	95% CI	*p* value
Pathology	.179	4.791	0.596–38.513	.141
No. of dissected LNs	.157	1.966	0.397–9.740	.408
Pathologic T stage	.193	2.965	1.065–8.252	.037*
LVI	.015	5.845	1.179–28.984	.031*

Bold indicates the significant values (*p < 0.05)

## Discussion

Gastric cancer is one of the most common malignant tumors in many countries around the world. Although its morbidity and mortality have declined in recent decades, due to the aging population, the number of new cases is still high every year [13]. Because patients with early gastric cancer have no specific clinical manifestations, except for South Korea and Japan, other countries in the world do not routinely carry out gastric cancer screening, and most gastric cancers are diagnosed only when they have progressed. For locally advanced gastric cancer, D2 radical surgery is the standard procedure. However, due to various factors, such as technical conditions and surgical experience, not all cancer centers in China can perform standard D2 radical resection. Even with standard R0/D2 radical resection, the local recurrence rate reported by different studies can still be as high as 20–40% [14].

In the United States, the INT-0116 study established adjuvant chemoradiotherapy as the standard of care in patients who have undergone curative resection for high-risk gastric adenocarcinoma [6]. However, the adoption of this regimen has been somewhat limited in China. Reasons include inadequate node dissection (only 10% had a D2 dissection) and the high morbidity rate observed, with 17% of patients in the INT-0116 study who discontinued adjuvant therapy because of toxicities. In China, the Guidelines for the Diagnosis and Treatment of Gastric Cancer from the National Health Commission, which has been updated three times since 2010, explicitly emphasize the necessity of adjuvant chemoradiotherapy for high-risk locally advanced gastric cancer in one of the following three situations: the patient received R1 or R2 operation; the patient received D0 or D1 resection, with T3–4 disease or conformed metastasis in perigastric LNs according to AJCC 8th staging; or the patient received R0/D2 resection, with postoperative histologically conformed metastasis in perigastric LNs. Thus, the objectives of this retrospective study were to report the efficacy and toxicities of such an approach, characterize the patterns of failure, and investigate the relationship between clinicopathologic factors and recurrence in patients after ≥ D1+ resection and adjuvant chemoradiotherapy. The current analysis offers important implications in terms of adjuvant chemoradiotherapy in patients after ≥ D1+ dissection. First, 3-year OS and DFS rates of 65 and 61%, respectively, and 5-year OS and DFS rates of 59.5 and 55.6%, respectively, were reported in this study, indicating favorable outcomes for patients in the INT-0116 study; however, these outcomes are slightly inferior to those in the ARTIST trial [6, 9, 15]. These results might be due to the earlier stage and more aggressive lymph node dissection in the ARTIST study in Korea. Only 40% of the patients enrolled in the ARTIST trial had stage III disease, but in this study cohort, 66.6% of the patients had stage III disease, which is a universal phenomenon in China due to the lack of a national screening project. Second, local or regional recurrence was a rare event occurring in only 3.2 and 9.0% of all patients; these rates are much lower than those from previous analyses from Western countries and are similar to the outcomes in Korea, offering further evidence that postoperative chemoradiotherapy might be useful for optimizing locoregional control. In the current study, distant metastasis was the most common pattern of failure. Furthermore, previous studies investigating patterns of failure in patients after adjuvant chemotherapy alone demonstrated that the incidence of locoregional relapse varied from 7.8 to 29.3%. According to reports from China, locoregional recurrence occurred in 32.4% of all treated patients [5]. Although the constitution of the failure pattern was similar, the incidence of locoregional recurrence in the current study was slightly lower than those in previous studies, suggesting the potential benefit of chemoradiotherapy for local control. Compared with the INT-0116 and Korean studies, we observed a lower incidence of gastrointestinal and hematological acute grade 3 or 4 adverse effects [15].

**Fig. 5:**
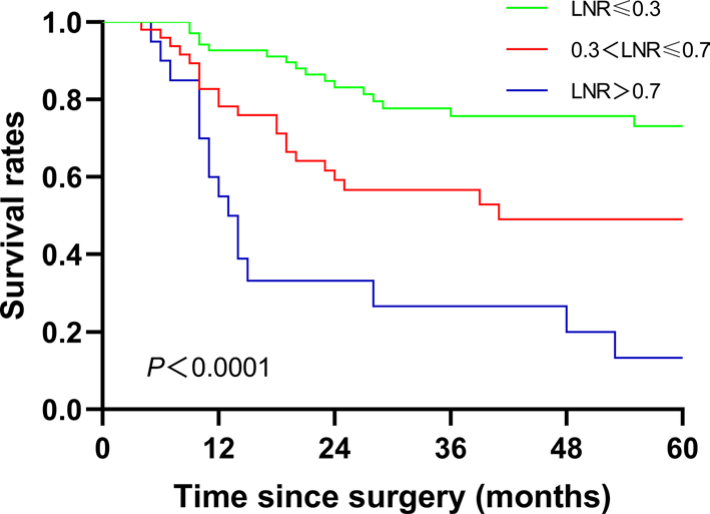
Kaplan–Meier estimate of OS of patients with different LNRs

LNR stands for the ratio between pathological metastatic lymph node number and total number of retrieved nodes. In recent years, many clinical studies have indicated that the LNR could be a significant prognostic factor for gastric cancer patients after surgery and is even considered to have better prognostic value than TNM staging [15–21]. Our results are consistent with those of previous retrospective studies reporting that the LNR is an effective prognostic tool after curative gastrectomy in addition to limited LN dissection (Fig. 5). Although the guidelines for gastric cancer treatment indicate that at least 15 LNs should be removed, it is important to emphasize that the number of LNs removed might vary among surgeons depending on patient selection, the extent of LN dissection, and the number of LNs examined by the pathologists. In fact, the LNR has also been proposed as a prognostic tool related to LN metastases. The principle behind the LNR arose from doubt over the impact of extended LN dissection on prognosis and discussions over whether improvements in nodal staging might be completely responsible for this effect. The fact that the chances of finding a positive LN were higher for more extensive LN dissection than for limited surgery led the authors to postulate that the significance of the LN stage would vary among the patients depending on the number of LNs removed.

Compared with the INT-0116 and ARTIST studies, we observed a lower incidence of gastrointestinal acute grade 3 or 4 adverse effects [15, 22], partly because IMRT was used. Several studies have proven that IMRT is superior to two- or three-dimensional radiotherapy, as it provides a more consistent dose to the PTV and accordingly minimizes the risk of toxicity [23–25].

## Conclusions

In summary, postoperative concomitant IMRT and chemotherapy are well tolerated in the Chinese population, with acceptable toxicities and encouraging tumor locoregional control and long-term survival for locally advanced gastric cancer patients after ≥ D1+ resection. LNR can be used as an important prognostic indicator for gastric cancer patients with ≥ D1+ resection and adjuvant chemoradiotherapy.

## Data Availability

The datasets used during the current study are available from the corresponding author on reasonable request.

## Abbreviations

DFS: Disease-free survival
LRRFS: Locoregional recurrence-free survival
CRT: Chemoradiotherapy
IMRT: Intensity-modulated radiation therapy
LN: Lymph node
M0: No clinical evidence of distant metastasis
OS: Overall survival
LFFS: Local failure-free survival
RFFS: Regional failure-free survival
PFFS: Peritoneal failure-free survival
DFFS: Distant failure-free survival
LRFFS: Locoregional failure-free survival
TNM: Tumor-node-metastasis
LVI: Lymphovascular invasion
PNI: Perineural invasion
LNR: Lymph node ratio
